# Microbial Volatile Organic Compounds: Insights into Plant Defense

**DOI:** 10.3390/plants13152013

**Published:** 2024-07-23

**Authors:** Vicente Montejano-Ramírez, José Luis Ávila-Oviedo, Francisco Javier Campos-Mendoza, Eduardo Valencia-Cantero

**Affiliations:** Instituto de Investigaciones Químico Biológicas, Universidad Michoacana de San Nicolás de Hidalgo, Edifico B3, Ciudad Universitaria, Morelia 58030, Mexico; 0678380c@umich.mx (V.M.-R.); 1355646g@umich.mx (J.L.Á.-O.); 1700143a@umich.mx (F.J.C.-M.)

**Keywords:** bacterial VOCs, fungal VOCs, yeast VOCs, microalgae VOCs, oomycete VOCs, effector-triggered immunity, pattern triggered immunity

## Abstract

Volatile organic compounds (VOCs) are low molecular weight molecules that tend to evaporate easily at room temperature because of their low boiling points. VOCs are emitted by all organisms; therefore, inter- and intra-kingdom interactions have been established, which are fundamental to the structuring of life on our planet. One of the most studied interactions through VOCs is between microorganism VOCs (mVOCs) and plants, including those of agricultural interest. The mVOC interactions generate various advantages for plants, ranging from promoting growth to the activation of defense pathways triggered by salicylic acid (systemic acquired resistance) and jasmonic acid (induced systemic resistance) to protect them against phytopathogens. Additionally, mVOCs directly inhibit the growth of phytopathogens, thereby providing indirect protection to plants. Among the current agricultural problems is the extensive use of chemicals, such as fertilizers, intended to combat production loss, and pesticides to combat phytopathogen infection. This causes problems in food safety and environmental pollution. Therefore, to overcome this problem, it is important to identify alternatives that do not generate environmental impacts, such as the application of mVOCs. This review addresses the protective effects of mVOCs emitted by microorganisms from different kingdoms and their implications in plant defense pathways.

## 1. Introduction

Volatile organic compounds (VOCs) are small molecules of low molecular weight, generally <300 Da, because of their high vapor pressure and low boiling points. They tend to be lipophilic and easily disappear between air and water. VOCs are easily identifiable as many of them are odorous molecules [1].

**Figure 1:**
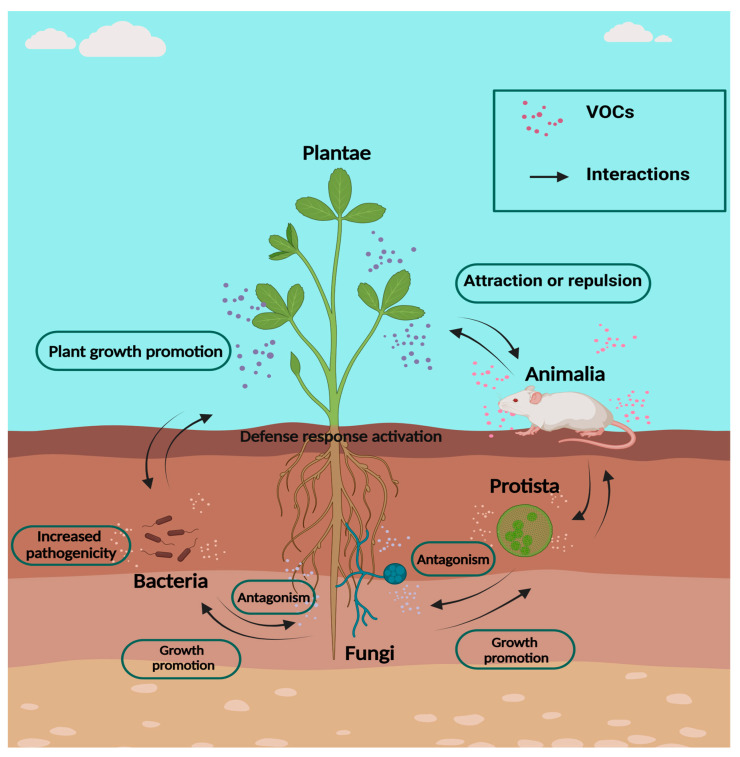
The interaction between living beings occurs not only physically but also through the emission of VOCs. VOCs maintain interaction within and between kingdoms both above and below ground. This interaction is important because it helps to structure ecosystems and allows organisms to respond quickly to the presence of neighboring beneficial or harmful organisms.

Virtually all organisms, including bacteria, fungi, plants, animals, and humans, produce VOCs whose function in distant chemical interactions >20 cm plays an important role between kingdoms (Figure 1), which participate in the structuring of life on our planet [2]. This set of volatile metabolites produced by different organisms is called the volatilome and represents a part of the metabolome [3]. In ecosystems, VOCs have important functions because they are used by flowers to attract pollinators, thereby facilitating fruit and seed production [4]. Additionally, these compounds have been exploited by humans in industry, applying them as aromatic components in perfumes, shampoos, and soaps [5]. They are commonly used in the food industry to produce aromas for cheese, wine, and the fermentation of some foods [6,7,8]. In human health, VOCS are useful for repelling or attracting disease vectors, such as mosquitoes. These compounds can be odorous, such as oils from citronella and geranium, or non-odorous synthetics, such as *N*, *N*-diethyl-meta-toluamide (DEET) [9].

Other VOCs are produced in the petrochemical industry, refineries, and vehicle emissions, which contribute to pollution in urban areas [10,11]. Therefore, when VOCs are emitted by various anthropogenic activities, they react with various atmospheric oxidants to form alkyl (R), alkyl peroxyl (RO_2_), and alkoxyl (RO) radicals, and then they participate in the nitrogen oxide (NO_x_) and HO_x_ cycles, contributing to O_3_ and aerosol formation [12]. This increases the levels of photochemical O_3_, which causes pollution in many urbanized areas, thereby affecting human health and air quality [13].

The advantage of VOCs, mainly microbial VOCs (mVOCs), is their ability to promote plant growth since they activate mechanisms in plants, which provide them with nutrients and minerals, in addition to triggering seed germination [14]. This promoting effect is because these compounds can modify the physiology of plants, as well as hormonal pathways, which increase biomass and production yield through improving plant characteristics such as the leaves and root, morphological changes in flowers, and an increase in fruit and seed production [15].

The most prevalent mVOCs are derived from fatty acids, including alcohols, alkanes, alkenes, aromatic compounds, and compounds containing sulfur, nitrogen, and terpenoids [16]. In agriculture, communication and interaction in the soil ecosystem between plants and microbes play a crucial role in structuring soil bacterial communities, which ultimately determine the growth of the plant and its health, which is reflected in agricultural productivity [17]. 

In addition, mVOCs and plant roots can travel long distances between the soil matrix and have the ability to mediate the interaction between separate metarhizobiomes in the rhizosphere, which gives rise to a wide range of interactions between plant rhizobiome networks, which have positive effects on the stability of the plant rhizobiome. The metarhizobiomes have species diversity and functional redundancy, acting as a population reservoir, significantly changing soil conditions, for example, during crop rotation [18].

Among the current agricultural problems is the extensive use of chemicals, such as fertilizers, intended to combat production loss triggered by climate change due to the increase in drought events, which causes problems in food safety and generates environmental pollution. Another problem is that climate change favors the dispersion of phytopathogens in larger geographic areas where new hosts are found, which makes epidemics more frequent [19]. Monocultures also favor the appearance of epidemics caused by fungi, bacteria, nematodes, and viruses [20]. To deal with this problem, the application of VOCs is proposed, because they allow defense mechanisms to be activated between distant plants, in addition to activating “priming” against future threats, which is known as “green vaccination” [21]. The defense mechanisms activated by VOCs defend plants against insects and fungi, which are two of the biotic factors that cause the most agricultural losses, as these two organisms represent two of the most widely distributed groups in nature [22]. 

Among the pool of VOCs that activate defense responses against biotic factors in plants, mVOCs are among the most studied because of their diverse metabolic origins and chemical structures. Plants exposed to mVOCs activate defense pathways throughout the entire plant, even in areas that are not exposed to a particular plant pathogen. 2R,3R-butanediol is an mVOC whose protective effect has been evaluated in the field and protects pepper plants exposed to viral infections by inducing defense-related genes. In addition to 2R,3R-butanediol, other mVOCs such as alkane tridecane induce the expression of defense genes and protect against symptoms caused by *Pseudomonas syringae* in *Arabidopsis thaliana*. Indole, another compound found in relatively abundant amounts in a blend of mVOCs, has a protective effect against herbivores and functions as a signal to warn neighboring plants and prepare them against future attacks by activating their defense systems. Despite the various studies on the induction of activated defense in plants by the presence of mVOCs, the receptors and regulatory genes, as well as the signaling pathways involved in their recognition, are still unknown; however, these compounds penetrate the cell membrane easily, and once inside the cell, they cause differential gene expression, affecting the physiology and behavior of the target organisms [14,23].

Therefore, exploitation of the potential of VOCs and mVOCs as promising strategies for sustainable biocontrol to replace pesticides and fertilizers has increased in recent agricultural systems [24].

Considering that all organisms produce VOCs, and given the importance that these currently represent in improving the resistance of crops, the present review focuses on the compilation of information on the effect of VOCs of different microbial origins on the establishment of plant defense.

## 2. Plant Defense Strategy

Plants interact with both biotic and abiotic factors in their environment. In this interaction, the plant also faces the presence of phytopathogens and herbivorous insects or those that feed on vascular tissues. Because plants are sessile organisms, their defense strategies are determined by the activation of their molecular mechanisms. During pathogenesis, phytopathogens produce molecules with highly conserved structures, such as lipopolysaccharides in the outer membrane of Gram-negative bacteria, chitin in fungal cell walls, flagellin, siderophores, and antibiotics, which are perceived by plants through proteins called pattern recognition receptors (PRRs) to activate pattern-triggered immunity (PTI) [25]. PTI is characterized by the activation of mitogen-activated protein kinases (MAPKs), the production of ethylene (ET), and an increase in the production of reactive oxygen species (ROS), to stop the internal progress of pathogens [26]. Phytopathogens can bypass the first line of defense (PTI) by inhibiting it through the production of effector proteins. 

In the presence of effectors, plants activate their perception through R proteins to trigger a second line of defense, effector-triggered immunity (ETI), which is focused on programmed cell death (PCD) and characterized by a high production of ET [27].

Additionally, during the first line of defense (PTI), there is an increase in the synthesis of phytohormones to activate two accessory pathways: induced systemic resistance (ISR) and systemic acquired resistance (SAR) [28]. ISR focuses on defense against necrotrophic organisms as well as the population regulation of plant growth-promoting rhizobacteria (PGPRs) and chewing insects. However, in the SAR response, plants generate resistance to biotrophs and insects that feed on the phloem [29,30].

The ISR pathway is characterized by an endogenous increase in jasmonic acid (JA), while SAR is characterized by an increase in the concentration of salicylic acid (SA) [31]. JA increases the expression of *Plant Defensin 1.2* (*PDF1.2*) through the transcription factor ethylene-responsive factor (ERF), which activates the ISR defense pathway [32]. In contrast, expression of the *pathogenesis-related 1* (*PR1*) gene via the transcription factor non-pathogenesis-related 1 (NPR1) is activated by SA, leading to the establishment of an SAR response [33].

## 3. Importance of Bacterial Volatile Organic Compounds in Plant Health

Bacteria have been an important subject of study in agricultural production. Some soil bacteria enhance plant development through direct or indirect mechanisms [34]. Direct mechanisms include the production of auxin [35], ACC deaminase [36], cytokinin [37], nitrogen fixation [38], and phosphorous solubilization [39]. Indirect mechanisms include quorum sensing competition for nutrients and the production of hydrogen cyanide, cell wall-degrading enzymes, and antibiotics (VOCs) [40].

**Figure 2:**
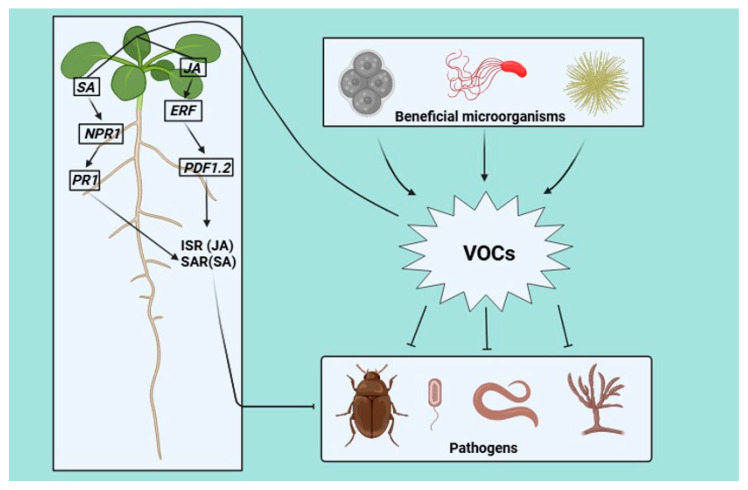
The protection established by the emission of VOCs of bacterial and fungal origin is one of the most studied, particularly microorganisms and compounds that combat pathogens such as insects, bacteria, fungi, and nematodes. Additionally, these VOCs activate defense responses in plants through the increment in JA and SA phytohormone concentration, therefore the activation of *ERF* and *NPR* transcription factors and the subsequent induction of *PDF1.2* and *PR1* defense genes expression to finally trigger the ISR and SAR pathways, thus generating indirect protection, which is related to the plant’s own chemical and molecular mechanisms. Lines with arrows indicate activation and broken lines indicate inhibition.

Bacteria produce numerous volatile compounds. Bacterial VOCs (bVOCs) play an important role in the interactions between plants and phytopathogens (Figure 2) [41]. One of the main functions of bVOCS is to act with bacterial elicitors to activate plant defense mechanisms, which are also known as microbe-associated molecular patterns (MAMPs). However, the mechanisms underlying the interaction of bVOCs with plants and phytopathogens have not been fully explored [42]. This is because the bVOC profile changes depending on the growth conditions of the producing bacteria and subsequently, the biotic or abiotic stresses to which they are subjected. 

Furthermore, bVOCs have different chemical natures (alcohols, aldehydes, alkenes, alkynes, benzenes, esters, heterocycles, ketones, sulfides, and terpenoids) [43]. Consequently, depending on the chemical nature, they are recognized by the plant’s PRRs and induce defense. A wide repertoire of signaling pathways is involved in the activation of plant defense by bVOCs, such as SA, JA, ET, and other plant hormones, such as auxin (IAA), brassinosteroids, and oxylipins [23,44,45,46].

Here, we review select examples of the ability of VOCs produced by bacteria to activate plant defense against phytopathogens. Since fungi have been economically relevant throughout history because they affect crops of agricultural interest [47], bVOCs have been reported to reduce the damage caused to plants through the induction of a systemic response. The compound 2,3-butanediol (2,3-BD) at a concentration of 250 μmol/L induced *Agrostis stolonifera* resistance against *Rhizoctonia solani*. The principal mechanism was an increase in phenol, flavonoid, and lignin levels, similar to IAA levels. This can positively interact with JA signaling and induce higher levels of phenylalanine ammonia-lyase (PAL), chalcone isomerase, and 4-coumarate activity [48]. 

The compound dimethyl disulfide (DMDS) produced by *Bacillus cereus* significantly protects tobacco and corn plants against *Botrytis* [49]. Additionally, this compound induced the overexpression of pathogenic genes (*PR1*) and (*PR5*) related to the growth and defense of tomatoes against the pathogen *Sclerotinia minor* [50]. Other bVOCs, such as 2-ethyl-5-methylpyrazine and dimethyl disulfide, produced by *Streptomyces setonii* showed strong antifungal activity against *Ceratocystis fimbriata* in vitro and increased the content of the antioxidant enzymes PAL, polyphenol oxidase (PPO), and total flavonoids in sweet potato [51].

Among bacteria exist phytopathogens that can cause diseases in crops worldwide, and consequently, have negative effects on agriculture. bVOCs play a significant role in the control of phytopathogens; for example, *Bacillus* spp. produce 3-hydroxy-2- butanone (acetoin) and 2,3-butanediol, which function as elicitors to activate ISR in *A. thaliana* through the ethylene pathway and are independent of the SA or JA signaling pathways [52]. 

Additionally, the bVOCs produced by the endophytic *Pantoea* sp. and *Pseudomonas* sp. Bt85 upregulated the expression levels of defense genes *PR1* and *nucleotide binding site leucine-rich repeat* (*NBS-LRR2*) (SA and JA pathways, respectively) in sugar beetroot slices against *B. pumilus* Isf19 [53]. Other bacteria activate the systemic response in plants through other pathways; for example, the bVOCs of *B. subtilis* KA9 and *P. fluorescens* PDS1 significantly increased defensive enzyme activity and overexpressed the *antioxidant genes PAL*, polyphenol oxidase activity (*POD*), superoxide dismutase (*SOD*), *WRKYa*, phenyl ammonia lyase (*PAL1*), defensive (*DEF-1*), catalase 2 (*CAT-2*), lipoxygenase 2 (*LOX2*), nonexpressor of pathogen-related (*NPR1*), *WRKY40*, and *HSFC1* in chili plants against *Ralstonia solanacearum* [54]. Additionally, the elicitor effect of bVOCs produced by *B. subtilis* GB03 has been reported in the activation of cucumber plant defense against *P. syringae* pv. lachrymans through the JA signaling pathway (defense-related gene *CsLOX*) [55].

Nematodes are organisms that affect agronomic plant development. Because the bVOCs produced can also induce systemic protection against parasitic diseases, the compounds benzaldehyde and dimethyl disulfide produced by *Ochrobactrum pseudogrignonense* are highly efficacious in managing root-knowing nematodes [56]. The bVOCs 2-undecanone and ND methyl isovalerate produced by *B. atrophaeus* GBSC56 showed strong nematicidal activity against *Meloidogyne* in infested tomato plants. bVOCs trigger the ISR-positive regulation of defense-related genes (*PR1*, *PR5*, and *SlLOX1*), resulting in a reduction in root galls in infested plants [57]. 

The application of bVOCs in greenhouses and open spaces has important limitations, such as decreased survival and activity of introduced bacteria in the natural environment, lack of stability of target bVOCs, and rapid evaporation rate [58]. However, under greenhouse and open field conditions, 2,3-butanediol reduced the accumulation of different viruses (TYLCV, TSWV, and PepMoV) in *Capsicum annum* [L]. ‘Bukwang’, the effect, appears to be dependent on SA, JA, and ET [59]. Other potential compounds for field application are 3-pentanol and 2-butanone, which induce the systemic response in Cucumber plants against *P. syringae* pv. Lachrymans and the sucking insect aphid *Myzus persicae*, with upregulated CsLOX1 expression, which is a marker protein of the oxylipin pathway [60]. Additionally, 3-pentanol compounds produced by *B. amyloliquefaciens* modulate defense priming by inducing the expression of SA- and JA-related resistance markers (*CaPR1*, *CaPR2*, *CaPR4*, and *CaGLP1*) [61]. 

The application of bVOCs (Table 1) requires further investigation to develop new approaches for sustainable agricultural production and reduce the application of chemical compounds.

## 4. Role of Fungal Volatile Organic Compounds in Plant Defense Pre- and Post-Harvest

Fungi produce various compounds, including high-value metabolites, such as enzymes and VOCs; the latter are used for communication between fungal cells or interactions between fungi and different species, such as insects, plants, and other microbes [62]. Fungal VOCs (fVOCs) are used in different disciplines, such as aroma and flavor chemistry, building science, chemical ecology, hygiene, medical mycology, and plant pathologies [63]. One of the most notable functions of fVOCs is their ability to activate priming in plants and protect them from future pathogen or predator attacks (Figure 2) [64].

### 4.1. Trichoderma spp. Emitte fVOCs with Protective Effect against Fungal Diseases

Species such as *Trichoderma asperellum* and *T. harzianum* induce the priming of plants. In the case of *T. asperellum* T1, the effects of the fVOCs produced by this fungus on the growth of *Conyespora cassiicola* and *C. aeria*, two lettuce (*Lactuca sativa*) pathogens that cause leaf spot disease, were evaluated. The diameters of the colonies treated with fVOCs were smaller than those of the controls with inhibition rates of 61% for *C. cassiicola* and 41% for *C. aeria*. The direct protective effect of this fungus on lettuce plants was analyzed two weeks after exposure to fVOCs. The activities of the cell wall-degrading enzymes b-1,3-glucanase and chitinase were higher in lettuce exposed to fVOCs compared to controls. Additionally, using a commercial version of the latter, the dominant fVOCs were found to be fluoro (trinitro) methane and 2-phenylethanol, resulting in an antifungal effect similar to that of the fVOCs, which is why it was proposed to be responsible for the protective effect [65]. 

Other results highlight the ability of fVOCs from both *T. asperellum* and *T. harzianum* to protect *A. thaliana* (L) plants against *B. cinerea* infection by inducing the expression of the *VSP2* and *PDF2.1* genes related to the ISR defense pathway activated by JA [66]. Diverse plants can be protected by *Trichoderma* fVOCs. In a study on *Vitis vinifera* (L.), the protective effects of fVOCs produced by *T. asperellum* T34, *T. harzianum* T39, and *T. atroviride* SC1 against the fungus *Plasmopara viticola*, which causes downy mildew, were tested. Various fVOCs were identified using head space–solid-phase microextraction gas chromatography–mass spectrometry; however, those that showed an effect in reducing downy mildew were α-farnesene, cadinene, 1,3-octadiene, 2-pentylfuran, and 6-pentyl. 2H-pyran-2-one, particularly 6-pentyl-2H-pyran-2-one and 2-pentylfuran, increased callose accumulation and enhanced the modulation of defense-related genes. In addition, 6-pentyl-2H-pyran-2-one activated a hypersensitivity response after inoculation with *P. viticola* [44]. 

In addition to protecting against phytopathogenic fungi, fVOCs produced by *Trichoderma* also activate the SAR pathway in response to tobacco mosaic virus (TMV): specifically, 6-pentyl-α-pyrone (6PP), which was isolated from *T. koningii* CTX1172 (AUMC 11520). *Nicotiana tabacum* (L.) plants infected with TMV and treated with low concentrations of 6PP (10–30 μg mL^−1^) inhibited 10–60% of infection, while when using high concentrations (40 and 50 μg mL^−1^), 100% inhibition was achieved. Plants treated with 6PP showed an induction of *PR-a*, *PR-b*, and *PR-10*, genes key to the SAR pathway triggered by SA, from the first day of treatment; this induction was greater in the presence of TMV and 6PP and was maintained until the seventh day [67]. Therefore, fVOCs of the genus *Trichoderma* have been proposed as possible treatments to protect plants of agricultural interest from pathogenic fungi that cause agricultural and economic losses worldwide.

### 4.2. Endophytic Fungi Also Produce Antifungal fVOCs, the Case of Muscodor Genus

Certain species of the *Trichoderma* genus are well known for their protective capacity against phytopathogens through the emission of fVOCs; however, there are other species of fungi whose volatile compounds also protect plants against biotic stress. The *Muscodor* genus includes several endophytic fungal species characterized by producing extremely bioactive fVOCs [68]. *M. albus* produces antimicrobial fVOCs that control post-harvest diseases. *M. albus* fVOCs inhibit or kill numerous phytopathogenic fungi. Therefore, their effects on latent and physiologically active teliospores of the fungi *Tilletia horrida*, *T. indica*, and *T. tritici* which cause rice smut, wheat smut, and common wheat smut, respectively, were analyzed. The teliospores of the three fungi fumigated for 5 days with the fVOCs of *M. albus* were unable to germinate [69].

In contrast, *M. albus* fVOCs directly protect plants against *Fusarium sambucinum*, *Helminthosporium solani*, and *Pectobacterium atrosepticum*, which are pathogens of potato (*Solanum tuberosum* L.) that cause dry rot, silver scurf, and bacterial soft rot, respectively. *M. albus* fVOCs inhibit the growth of *F. sambucinum*, *H. solani*, and *P. atrosepticum*. In addition, biofumigation with *M. albus* significantly reduced dry rot and completely controlled silver scale [70]. Other species, such as *M. crispans*, an endophytic species of *Ananas ananassoides*, produce a mixture of fVOCs, including propanoic acid, 2-methyl-, methyl ester; propanoic acid, 2-methyl-; 1-butanol, 3-methyl-;1-butanol, 3-methyl-, acetate; propanoic acid, 2-me-thyl-, 2-methylbutyl ester; and ethanol, which is the most abundan [71]. These fVOCs have antibiotic properties and are effective against a wide range of phytopathogenic fungi like *Pythium ultimum*, *Phytophthora cinnamomi*, *S. sclerotiorum*, and *Mycosphaerella fijiensis*, as well as citrus pathogenic bacteria, *Xanthomonas axonopodis* pv. *citri*. [72]. These results suggest that the fVOCs of *M. albus* have potential applications in agriculture. Another endophytic species of the *Muscodor* genus is *M. brasiliensis* sp. nov., which emits phenylethyl alcohol, α-curcumene, and E (β) farnesene fVOCs and completely inhibits the phytopathogen *Penicillium digitatum*. It also protects post-harvest oranges against mold formation, observing a reduction of 77%, highlighting that these fVOCs are protectors in the storage and transportation of fruits [72]. 

Additionally, a characteristic citrus disease, citrus black spot (CBS), caused by *Phyllosticta citricarpa* affects the industry worldwide. fVOCs produced by endophytic fungi of these plants, specifically *M. sutura*, inhibit the growth of the phytopathogenic fungus [73]. The fVOCs of fungi of the *Muscodor* genus protect plants of agricultural, nutritional, and economic interest against species of phytopathogenic fungi that are important in their global production; in some cases, there is no difference between the protection provided by fungicides [74], which strengthens the idea of investing in techniques focused on sustainable agriculture and “green vaccination”.

### 4.3. fVOCs Provide Protection for Plants against a Wide Range of Phytopathogens

The protection provided by fVOCs can be extended to various microbial phytopathogens. In addition to fungi, fVOCs protect against bacterial attack. Fungal species such as *Cladosporium* sp. and *Ampelomyces* sp. emitted mixtures of fVOCs that significantly reduced the severity of infection by *P. syringae* pv. tomato and DC3000 (Pst.). From these fVOC mixtures, m-cresol and methyl benzoate (MeBA) showed the highest activity. q-PCR revealed that m-cresol activates both SA- and JA-dependent genes (*PR1*, *PR2*, *PR4*, and *PDF1.2*), whereas MeBA is mainly involved in the JA-dependent pathway and only partially in the SA pathway (*PR1*, *MYC2*, *PDF1.2*, and *VSP2*) [75].

Fungi of the *Daldinia* genus have broad biological activities in several areas of human health, including antimicrobial activity [76]. fVOCs not only protect plants against phytopathogenic fungi but also against other types of attackers, such as nematodes, specifically sedentary root-knot nematodes (RKNs), *Meloidogyne* spp., which are extremely polyphagous and exploit a wide range of hosts. The fVOCs of *D.* cf. *concentrica* controlled RKN *M. javanica* in vitro and in the greenhouse through bionematicidal activity in juvenile stages. In greenhouses, the application of fVOCs, mainly 4-heptanone, had the same effect as the mixture of fVOCs with a nematicidal activity of 90% in reducing viability [77]. *Daldinia* also stands out as a biocontrol because its fVOCs are active against various phyla of fungi, protect post-harvest fruits such as against fungal growth in nuts, and eliminate the infection of peanuts by *Aspergillus niger* [78].

### 4.4. Entomopathogenic Fungi Modify the Insect Herbivore Behaviors by fVOCs Emission

In addition, entomopathogenic fungi can protect plants from insects. The *Cosmopolites sordidus* beetle, whose larva feeds on the corm of the plant, generates tunnels inside and causes a reduction in crop yield; therefore, it is necessary to apply control techniques through natural mechanisms [79], such as the fVOCs emitted by *Beauveria bassiana* (Bb1TS11) and *Metarhizium robertsii* (Mr4TS04). A study identified 97 fVOCs, of which seven (styrene, benzothiazole, camphor, borneol, 1,3-dimethoxy-benzene, 1-octen-3-ol, and 3-cyclohepten-1-one) were selected due to their abundance and by its previous report as insect repellents. 3-cyclohepten-1-one was the best repellent against *C. sordidus*, which was followed by 1,3-dimethoxy-benzene [80]. In contrast, *B. bassiana* also protects against *Rhyncophorus ferrugineus* (Oliver), since it can infect and kill all stages of development of this beetle. However, through the fVOCs emitted by *B. bassiana*, females of *R. ferrugineus* can be repelled, and infestation in red palm *Cyrtostachys renda* (BLUME) can be avoided [81]. In a separate study, it was found that the fVOCs emitted by *B. bassiana* AS5, especially 3-methylbutanol, result in chemical changes in *Sorghum bicolor* plants. These changes cause deterrent responses in the feeding behavior of larvae of the lepidoptera *Spodoptera frugiperda* [82].

Considering that fVOCs protect against fungi, phytopathogenic bacteria, and pest insects for crops of agricultural importance, it is crucial to devise strategies for their application in crops, maintaining their effect for as long as possible, on the way to sustainable agriculture, and to reduce or eliminate the use of chemical pesticides.

## 5. Volatile Organic Compounds Emitted by Yeast Protect Plants against Phytopathogens 

The use of microorganisms, such as yeasts, to protect and control pathogen infections, both pre- and post-harvest, is a common strategy because these microbes have antagonistic effects against various pathogens, including fungi. Many yeasts are highly effective in protecting plants because of their high capacity to colonize many environments, and their presence is common in plant materials (Figure 2) [83].

In a study by Choinska et al. (2020) [84], the effects of three strains of yeast, *Pichia kudriavzevii* Kkp 3005, *P. western* Kkp 3004, and *Meyerozyma quilliermondii*/*M. caribbica* KKP 3003, were evaluated against phytopathogenic fungi such as *Mucor* SPP, *P. expansum*, *A. flavus*, *F. cereals*, *F. poae*, and *B. cinerea*. Through direct contact, it was observed that the evaluated yeasts had an inhibitory effect on all phytopathogens evaluated at a percentage of more than 50%. Through separate compartments, using divided boxes, the presence of (target volatile organic compounds) was analyzed. The main emitted yVOCs were ethyl esters of medium-chain fatty acids, phenylethyl alcohol, and acetate. After 5 days of incubation at 25 °C, the growth of the molds tested was distinctly inhibited (above 60%), except for *B. cinerea*, where the percentage of growth reduction was the lowest (11%). The antagonistic activity of yeasts was not restricted to phytopathogens but was also observed in different mold genera, which makes them potential microorganisms for application in biological control. Among the main fruits that suffer from post-harvest infections are strawberries, whose infections, especially those by fungi, cause losses in production. 

Other species of the genus *Pichia* also produce yVOCs with antifungal activity against *A. flavus* through 2-phenylethanol (2-PE). When the fungus *A. flavus* was treated with the yVOCs (2-PE) of *P. anomala* strain WRL-076, spore germination and aflatoxin production were inhibited through a 10,000-fold inhibition of the genes involved in fatty acid biosynthesis [85]. Considering the agricultural importance of *A. flavus*, whose infection includes corn, cotton, peanuts, and tree nuts, and also in many dried fruits and spices, causing great economic losses worldwide, the effects of 2-PE on *A. flavus* suggest that 2-PE can be an effective biocontrol agent against it [86].

Traditionally, fungal infection in strawberry (*Fragaria x ananassa* Duchesne ex Weston) has been combated through the use of chemical fungicides [87], which accumulate in soils and agricultural products. Therefore, the use of yVOCs emitted by the antagonist yeast *Hanseniaspora uvarum* is a harmless alternative to the environment and promotes the resistance and quality of strawberries. Strawberries fumigated with *H. uvarum* yVOCs showed an increase in the content of methyl caproate (5.8%), methyl octanoate (5.1%), and methyl caprylate (10.9%) in post-harvest cold storage, which are compounds involved in improving the flavor of the strawberry. It also increased the activity of key defense enzymes, such as CAT, SOD, APX42, PPO, and PAL6, which was also maintained at the genetic level with an induction in the expression of the genes that encode these enzymes (*CAT*, *SOD*, *APX42*, *PPO*, and *PAL6*) [88]. VOCs produced by yeasts *P. kudriavzevii* KKP 3005, *P. occidentalis* KKP 3004, and *M. quilliermondii*/*M. zyma caribbica* KKP 3003 did not have an inhibitory effect on the growth of *B. cinerea* [84]; however, another species of the genus *Hanseniaspora*, specifically *H. opuntiae*, isolated from *Opuntia ficus-indica* rot, inhibited the growth and number of spores of this fungus. In addition, the direct interaction of *A. thaliana* plants with yeast cells protects the plants against the fungus through independent pathways to auxins, ethylene, JA, or SA, because the effect is maintained despite the gene mutation pathways. These effects are attributed to the yVOCs emitted by yeast, among which 3-methyl-1-butanol and ethanol are the most abundant [89]. 

To date, studies evaluating the defense triggered by yeast volatiles have focused on protection against fungi (Table 2), owing to the importance of these volatiles due to their post-harvest appearance, which is why their application could be considered before the storage of various fruits. Additionally, the effect of these VOCs on the behavior of insects and phytopathogenic bacteria is a topic for further study.

## 6. Microalgae Volatile Organic Compounds as Novel Plant Protection Mechanism

Within the framework of sustainable agriculture, microalgal biomass, as well as the agrochemical compounds derived from it, are novel tools designed to protect plants in addition to promoting plant growth. The above effects are attributed to the presence of biomolecules, such as soluble amino acids, micronutrients, polysaccharides, phytohormones, and other signaling molecules present in the biomass of microalgae [90]. 

**Figure 3:**
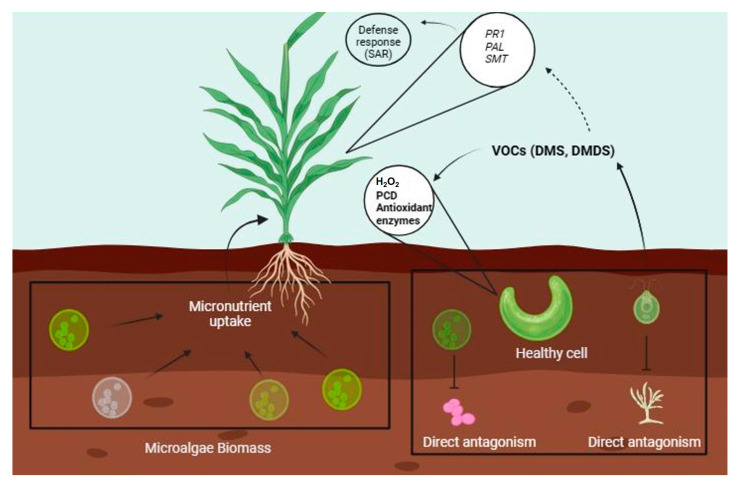
The use of VOCs from microalgae is a novel issue in stimulating plant growth and in the direct and indirect protection of plants. Microalgae are used as biomass or fertilizers to promote plant growth in addition to their direct antagonism against phytopathogens. However, studies have highlighted the ability of VOCs emitted by microalgae to activate defense responses in plants due to the induction of gene expression. The defense response is also activated in other neighboring microalgae not exposed to pathogens. Lines with arrows indicate activation and broken lines indicate inhibition.

The most common way to use microalgae is through the use of biomass and biofertilizers [91]. Microalgae combat pathogens by direct antagonism or indirectly, and once plants detect pathogens, they promote the activation of their defense pathways [92]. Plant defenses are activated by polysaccharides produced by microalgae, which function as elicitors (Figure 3) [93]. In *S. lycopersicum* (L.), plants injected with 0.2 mg mL^−1^ of crude extract of polysaccharides from four strains of green microalgae (*Chlorella vulgaris*, *C. sorokiniana*, *Chlamydomonas reinhardtii*, and *Dunaliella salina*) showed an increase in β-1,3-glucanase activity increased as well as the activity of enzymes involved in the synthesis of SA (such as PAL) and JA (such as lipoxygenase) and antioxidant enzymes (APX, POD, and CAT), which are involved in establishing defense in plants for pathogen cell wall breakdown and in the synthesis of phytohormones that activate SAR or ISR [94].

A little-explored application of microalgae involves the use of volatile organic compounds (maVOCs). maVOCs have diverse applications in biochemistry, metabolism and physiology, ecology, and environmental applications. They regulate predator–prey interactions and can be used as predictors of pond collapse owing to the presence of predators [95]. The application of maVOCs for the protection of plants against pathogens is less explored. However, there is a precedent that highlights their protective capacity. Zou et al. (2011) [96] demonstrated that when the microalga *C. reinhardtii* is subjected to acetic acid stress, the production of reactive oxygen species (H_2_O_2_) is triggered, leading to programmed cell death. 

When maVOCs are applied to healthy cells, the cell density is reduced, and the activity of antioxidant enzymes increases, which is a defense response against pathogens. *C. reinhardtii* responds similarly when exposed to maVOCs from cells grown under NaCl and NaCO_3_ stress [97]. Among the various maVOCs produced by microalgae, some contain sulfur, such as dimethyl sulfide and dimethyl disulfide [95], which in orange plants *Citrus sinensis* (L) trigger antiherbivory responses by inducing the expression of SA genes such as *PAL*, *salicylate-O-methyl transferase* (*SMT*), and *PR1* as well as an increase in the activity of defense-related enzymes PAL, PPO, and POD [98].

The application of maVOCs is poorly explored. Moreover, to date, the interaction between microalgae and plants has been studied through extracts or biomass, making this field open for further research.

## 7. Oomycetes as an Emerging Mechanism for Plant Protection

Oomycetes are aquatic molds and fungal-like eukaryotes that are classified as stramenopiles and are phylogenetically grouped with diatoms and brown algae [99]. They cause diseases in agriculture and aquaculture, which makes them problematic in terms of food security. In addition, they cause economic losses and damage to ecosystems [100]. 

Phytopathogenic species are those belonging to the genus *Phytophthora*, such as *P. infestans*, *P. palmivora*, and *P. ramorum*, which cause Irish potato famine, black cocoa pods, and sudden oak death, respectively [101]. Although the importance of oomycetes lies in their phytopathogenic capacity, some species parasitize fungi and other oomycetes that infect plants. *P. oligandrum* uses a process of degradation of the cell wall of its prey, using cellulases and chitinases [102,103]. *P. oligandrum* is a non-phytopathogenic oomycete that promotes plant growth either by producing auxins or by inducing resistance by elicitors [104,105].

**Figure 4:**
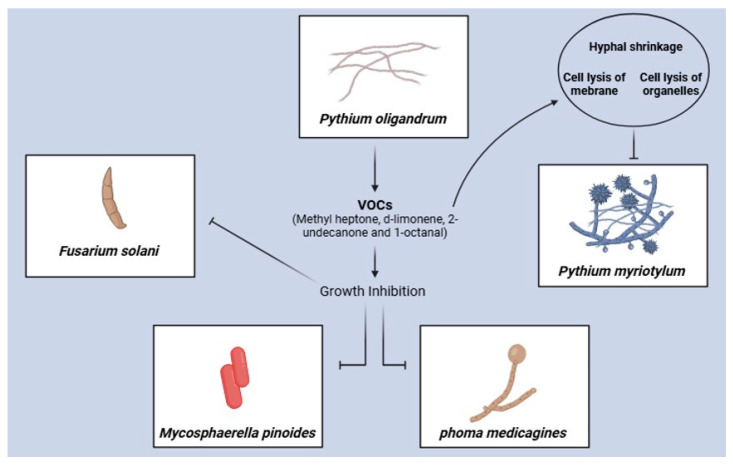
Although most oomycetes of the *Phytium* genus are phytopathogenic, *P. oligandrum* is an exception, which is known to disperse in plant roots and protect them against phytopathogens such as fungi and other oomycetes. A few studies have evaluated the effect of VOCs emitted by oomycetes on plant protection; however, oVOCs such as methyl heptenone, d-limonene, 2-undecanone, and 1-octanal have been proposed to be responsible for inhibiting pathogen growth and inhibiting *P. myriotylum* infection in ginger plants.

One of the first studies involving the activity of VOCs emitted by oomycetes (oVOCs) *P. oligandrum* dates back to 1991 [106], which showed that these compounds inhibit the growth of fungi (Figure 4), such as *F. solani*, *Phoma medicaginis* (syn. *Ascochyta medicaginicola*), and *M. pinodes* (syn. *Didymella pinodes*), which is a pathogen of peas. Sheihk et al. (2023) [107] evaluated the effect of oVOCs produced by *P oligandrum* to investigate whether they contribute to antagonism against the oomycete *P. myriotylum*. The oVOCs emitted by *P. oligandrum* inhibited the growth of *P. myriotylum* by 80% and the zoospore levels by 60%. Among the 23 oVOCs identified, methyl heptenone, d-limonene, 2-undecanone, and 1-octanal were potent inhibitors of *P. myriotylum* growth. Exposure to oVOCs led to hyphal shrinkage and the cell lysis of membranes and organelles of *P. myriotylum*. Previous data show that oVOCs are another mechanism linked to the parasitism of *P. oligandrum* in addition to the widely reported hydrolytic enzymes.

Sheikh et al. (2023) [108] investigated the effects of VOCs emitted by *P. oligandrum* on both plant growth and disease suppression in *N. benthamiana* (DOMIN.), showing an increase in biomass through 3-octanone and hexadecane. oVOCs also increase shoot and root growth in ginger plants. Additionally, transcriptomic analysis was performed in ginger plants, in which a greater expression of genes related to hormones that promote plant growth, such as auxin, zeatin, and gibberellic acid, was observed. Finally, regarding defense mechanisms, ginger leaves exposed to VOCs showed lower levels of infection by *P. myriotylum*.

As oomycetes are important for their phytopathogenic capacity, there is little information regarding the species that promote plant growth or protect plants against biotic stress (Table 3). To date, the most studied species for these capacities is *P. oligandrum*, which can be exploited for sustainable agriculture.

## 8. Conclusions

Agriculture is one of the most important human practices worldwide in terms of economic and nutritional issues and is, therefore, one of the most exploited. Consequently, agriculture generates changes in land use, contamination of water and soil with fertilizers and pesticides of chemical origin, negative effects on human health, and economic losses. One strategy to avoid using chemical compounds is to use microorganisms that promote plant growth, because many of these also protect plants against pathogens of agricultural importance, which generally correspond to phytopathogenic fungi. Beneficial microorganisms also produce VOCs, which are primarily responsible for promoting growth effects and activating defense pathways in plants. In a scenario focused on sustainable agriculture, the application of pre- and post-harvest VOCs is an alternative to combat the environmental effects of the excessive use of chemical compounds in agriculture. Additionally, considering the effectiveness of the VOCs of microorganisms from different kingdoms in inhibiting the growth of phytopathogens, the wide variety of species remaining must be explored, and effective microbial consortia must be developed to cover both growth and defense aspects in plants of agricultural interest.

## Figures and Tables

**Table 1:** List of bacterial volatile organic compounds and their known functions.

Organism	mVOC	Function	Ref. ^a^
Diverse rhizobacteria	2,3-butanediol (2,3-BD)	Resistance against *R. solani* and protects against different viruses	[48,59]
*B. cereus*, *Pseudomonas*, *Serratia*, and *Stenotrophomonas*	Dimethyl disulfide	Induction of *PR1* and *PR5* genes	[49,50]
*S. setonii*	2-ethyl-5-methylpyrazine and dimethyl disulfide,	Antifungal activity against *C. fimbriata* increased PAL and PPO enzymes	[51]
*Bacillus* spp.	3-hydroxy-2- butanone (acetoin) and 2,3-butanediol	Activation of ISR defense pathway	[52]
*Pantoea* sp. and *Pseudomonas* sp.	bVOCs blend	Upregulation of *PR1* and *NBS-LRR2* defense genes	[53]
*B. subtilis* KA9 and *P. fluorescens*	bVOCs blend	Upregulation of *PAL*, *POD*, *SOD*, *WRKYa*, *PAL1*, *DEF-1*, *CAT-2*, *LOX2*, *NPR1*, *WRKY40*, and *HSFC1* defense genes.	[54]
*B. subtilis* GB03	bVOCs blend	Activates defense against *P. syringae* pv.	[55]
*O. pseudogrignonense*	Benzaldehyde and dimethyl disulfide	Efficacious in managing *M. incognita*,	[56]
*B. atrophaeus* GBSC56	2-undecanone and ND methyl isovalerate	Nematicidal activity and upregulation of *PR1*, *PR5* and *SlLOX1 genes*	[57]
Many microorganisms	3-pentanol and 2-butanone	Protects against *P. syringae* pv. Lachrymans and *M. persicae*	[60]
*B. amyloliquefaciens*	3-pentanol	Induction of *CaPR1*, *CaPR2*, *CaPR4*, and *CaGLP1* defense genes.	[61]

^a^ Reference.

**Table 2:** List of fungi and yeast volatile organic compounds and their known functions.

Organism	mVOC	Function	Ref. ^a^
*T. asperellum* and *T. harzianum*	fVOCs blend	Inhibition of *C. cassiicola*, *C. aeria* and *B. cinerea.* Upregulation of *VSP2* and *PDF2.1* gene expression	[65,66]
*T. atroviride*	6-pentyl-2H-pyran-2-one and 2-pentylfuran.	Protects against *P. vitícola*	[44]
*T. koningii*	6-pentyl-α-pyrone (6PP)	Inhibition of tobacco mosaic virus and the upregulation of *PR-a*, *PR-b*, and *PR-10* SAR genes	[67]
*M. albus*	fVOCs blend	Inhibition of *T. horrida*, *T. indica*, and *T. tritici* spore germination. Protection against *F. sambucinum*, *H. solani*, and *P. atrosepticum*	[69,70]
*M. crispans*	Propanoic acid, 2-methyl-, methyl ester; propanoic acid, 2-methyl-; 1-butanol, 3-methyl-;1-butanol, 3-methyl-, acetate; propanoic acid, 2-methyl-, 2-methylbutyl ester; and ethanol	Antibiotic properties against *P. ultimum*, *P. cinnamomi*, *S. sclerotiorum*, *M. fijiensis*, and *X. axonopodis* pv. *Citri*	[71]
*M. brasiliensis*	Phenylethyl alcohol, α-curcumene, and E (β) farnesene	Inhibition of phytopathogen *P. digitatum*	[72]
*M. sutura*	fVOCs blend	Growth inhibition of *P. citricarpa*	[73]
*Ampelomyces* sp. and *Cladosporium* sp.	fVOCs blend mixture m-cresol and methyl benzoate (MeBA)	Reduces infection caused by *P. syringae* pv. tomato DC3000. Activation of SAR and ISR defense pathways	[75]
*D.* cf. *concentrica*	fVOCs blend	Bionematicidal activity against *M. javanica* Eliminates *A. niger* infection	[77,78]
*B. bassianana* and *M. robertsii*	Styrene, benzothiazole, camphor, borneol, 1,3-dimethoxy-benzene, 1-octen-3-ol, and 3-cyclohepten-1-one	Repellent against the *C. sordidus* beetle. Protection against *R. ferrugineus*	[80,81]
*P. kudriavzevii* Kkp 3005, *P. western* Kkp 3004, and *M quilliermondii*/*M caribbica* KKP 3003	Ethyl esters of medium-chain fatty acids, phenylethyl alcohol and acetate	Growth inhibition of *Mucor* spp., *P. expansum*, *A. flavus*, *F. cereals*, and *F. poae*.	[84]
*P. anomala* strain WRL-076	2-phenylethanol	Inhibition of spore germination and aflatoxin production in *A. flavus*	[85]
*H. uvarum*	yVOCs blend	Increased activity of defense enzymes: CAT, SOD, APX42, and PPO	[88]
*H. opuntiae*	3-methyl-1-butanol and ethanol	Growth inhibition and spore reduction in *B. cinerea*	[89]

^a^ Reference.

**Table 3:** List of microalgae and oomycete volatile organic compounds and their known functions.

Organism	mVOC	Function	Ref. ^a^
*C. reinhardtii*	maVOCs blend of stressed cells dimethyl sulfide and dimethyl disulfide	Activates defense responses in *C. reinhardtii* unexposed to stress Induction of the expression of defense genes *PAL* and *SMT PR1*	[97,98]
*P. oligandrum*	oVOCs blend and 3-octanone and hexadecane	Inhibits *F. solani*, *P. medicaginis*, and *M. pinodes* growth. Inhibits *P. myriotylum* growth and protects *N. benthamiana* against disease	[106,107,108]

^a^ Reference.

## Data Availability

No new data were created or analyzed in this study. Data sharing is not applicable to this article.
